# Travelling within the fetal gut: simple rules for an arduous journey

**DOI:** 10.1186/s12915-014-0050-z

**Published:** 2014-06-30

**Authors:** Qiling Xu, Tiffany Heanue, Vassilis Pachnis

**Affiliations:** 1 Division of Developmental Neurobiology, MRC National Institute for Medical Research, The Ridgeway, Mill Hill, London NW7 2NT, UK; 2 Division of Molecular Neurobiology, MRC National Institute for Medical Research, The Ridgeway, Mill Hill, London NW7 2NT, UK

## Abstract

The complex physiology of the gastrointestinal tract is regulated by intricate neural networks embedded within the gut wall. How neural crest cells colonize the intestine to form the enteric nervous system is of great interest to developmental biologists, but also highly relevant for understanding gastrointestinal disorders. A recent paper in *BMC Biology* addresses this issue with live imaging of gut explants from mouse embryos.

See research article: http://www.biomedcentral.com/1741-7007/12/23.

## Commentary

Neural crest (NC) cells constitute the archetypal migratory cell type of vertebrate embryos. They originate at the dorsal neural tube and, migrating along stereotypic pathways, colonize different regions of the embryo giving rise to diverse tissues, including neurons and glia of the peripheral nervous system (PNS), melanocytes, and musculoskeletal components of the head [[1]]. Most of these NC cell populations move as a cell collective to reach their final destination, but their specific migratory behavior can vary depending on the particular group of NC cells and the species. ‘Follow the leader’ chain migration, in which cells maintain relatively stable relations with the same neighbors, ‘run and tumble’, in which directional migration is followed by random and unpredictable cell movement, ‘mutual co-attraction’ and ‘contact inhibition’ are all examples of migratory behaviors that have been described for NC cells. Yet despite the recognition that enteric NC cells (ENCCs), which give rise to the enteric nervous system (ENS), perform one of the longest and most demanding journeys within the embryo, their migratory behavior is poorly characterized. By providing critical insight into the dynamic behavior of ENCCs during gut colonization, the recent paper in *BMC Biology* by Young and colleagues [[2]] goes a long way toward filling this gap in our knowledge.

The digestive system allows multicellular organisms to absorb useful nutrients, minerals and water, whilst preventing harmful substances and pathogenic microorganisms from entering into local tissues and the bloodstream. Key aspects of gastrointestinal function are under neural control that is provided by the bidirectional neurohumoral pathways of the gut-brain axis and the intrinsic ENS, which, unlike the rest of the PNS, can function independently of the central nervous system [[3]]. In vertebrates the ENS is made up of a vast number of neurons (in adult animals the gut contains as many neurons as the entire spinal cord) and four to five times as many glial cells. Enteric neurons and the majority of glial cells are packaged into interconnected ganglia that are organized into two layers, the outer myenteric and the inner submucosal plexuses, that extend as two concentric sleeves throughout the length of the gastrointestinal tract. Axons emerging from enteric ganglia crisscross the myenteric and submucosal plexuses and ultimately synapse onto neurons in other ganglia or make functional connections with extra-ganglionic tissues, such as smooth muscle, blood vessels and intestinal epithelium. Enteric neurons are highly diverse and the many subtypes identified on the basis of morphological, electrophysiological or molecular characteristics are distributed across the ganglionic network in a salt-and-pepper arrangement.

How does this complex neural system develop? Essentially, a small group of NC cell progenitors from the hindbrain invades the anterior end (foregut) of a rapidly extending cylindrical structure (gut tube) and gives rise to a vast network of neurons and glial cells that are distributed uniformly throughout its length [[4]]. To achieve this, the founder pool of ENS progenitors must advance along the gut while leaving behind sufficient numbers to colonize all the new areas they have occupied uniformly. In addition, the gut continues to expand long after the entire length of the intestinal wall has been colonized, thus demanding a continuous proliferation and reorganization of ENCCs. The current model for the uniform colonization of the gut mesenchyme posits that at the ENCC front some cells retain their migratory character and continue to advance caudally while others cease to migrate and stay behind in order to populate more rostral gut regions. Although this model accounts for the uniform colonization of the gut by NC cells, a sessile subpopulation of ENCCs has not been identified so far.

## ENCCs colonize the gut via ‘leapfrog’ migratory behavior

**Figure 1:**
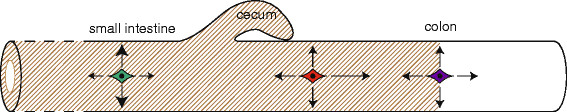
**Schematic showing the bias of ENCCs to be displaced along the rostrocaudal and circumferential direction.** The purple cell represents ENCCs at the migratory front of NC cells in the colon, which have equal probability to be displaced in all directions (equal size of arrows). The red cell represents more rostral ENCCs, which show a bias towards caudal displacement (caudally directed arrow is larger than the others) and allows them to overtake the leader cells ahead of them. The green cell represents pre-cecal ENCCs within the small intestine that migrate mostly circumferentially and show minimal caudal displacement (arrows along the long axis of the gut are smaller). Shaded area indicates the portion of the gastrointestinal tract that has been colonized by ENCCs.

The publication by Young and colleagues directly addresses some of these issues. Using an elegant approach that is based on the expression of a photoconvertible fluorescent reporter, the authors analyze the behavior of individual ENCCs and demonstrate that, in contrast to the prevailing view, cells that remain behind the advancing front continue to migrate. In fact, the average speed of migration of the most caudal ENCCs is only slightly higher than that of their more rostral counterparts. In addition, they demonstrate that the directionality of migration of any given ENS progenitor is unpredictable and that the migration of many solitary ENCCs can be described as a ‘random walk’. But given this drifting behavior of ENCCs, how do they manage to populate the gut in such an orderly manner and reproducible timeframe? The answer relies on the small bias of the leading ENCCs to remain within the segment of the gut they have just populated and the better chances of more rostral cells to advance caudally (Figure 1). Although the migratory pattern of any given ENCC is unpredictable, as a population follower cells behind the migratory front are likely to leapfrog the ones ahead of them and form the new leaders. This system of ‘rotating leadership’ seems to be very robust and highly effective in pushing ENCCs caudally. Interestingly, the work of Young and colleagues also suggests that this type of cell behavior is ultimately based on the ability of ENCCs to self-organize into cellular chains, rather than cell-intrinsic differences along the migratory sleeve. Perhaps ENCCs located at the very front are kept busy exploring, with their lamellipodia and filopodia, all the local options and the new space available to them, while more posterior cells find the opportunity to associate with prominent NC cell chains, which channel them to leading positions at the forefront.

The neuroectodermal networks of the gut remain in a state of flux long after the migratory front of NC cells has passed through a given segment of the intestine. This is clearly shown by experiments described by Young and colleagues which reveal that 24 to 48 hours after the arrival of the first NC cells in the developing small intestine, ENCCs continue to migrate extensively, although at this stage their trajectory is mostly circumferential with minimal rostrocaudal displacement. The predominantly radial migration of ‘older’ ENCCs could be imposed by changes in non-neuroectodermal tissues that accompany gut organogenesis, in particular the radial rearrangement of smooth muscle layers. The adoption by rostral ENCCs of a predominantly circumferential migration, which is ineffective in pushing cells caudally, is likely to explain why ENCCs must reach the end of the gut within a strict time frame: any delay in ENCC migration cannot be redressed later, and would make terminal gut regions neuron-free, thereby compromising the organ’s coordinated function. Nevertheless, the sustained ability of ENCCs to engage in local and radial migration means they can continue to add to and remodel enteric neural networks throughout embryogenesis and early life, when gut anatomy and physiology adjusts to feeding and the colonization of the intestine by microflora.

At present the only other well-studied example of ‘colonizing while migrating’ is the posterior lateral line primordium (pLLP), the placode-derived mechanosensory system that allows fish, and some amphibians, to detect even small changes in water movement over their bodies. Although some interesting parallels can be drawn between the migration of ENCCs and the migration of pLLP in zebrafish, whose transparent larvae make them particularly amenable to *in vivo* studies of cell migration, there are significant differences of cell behavior between the two systems. Thus, unlike the seemingly homogeneous population of ENCCs that ultimately are distributed throughout the gut wall, the pLLP remains as a well-defined and compact group of cells that maintains clear polarity along its length and deposits periodically from its trailing end rosette-like mechanosensory organ precursors [[5]]. In further contrast to the mammalian ENS, which continues to rearrange in an apparently unpredictable manner long after ENCCs have colonized the gut, pLLP maintains its organization and delivers an almost complete mechanosensory organ whose overall structure changes little following deposition. Interestingly, despite initially dispersing to form a diffuse ENS, ENCCs in zebrafish are initially organized into two compact columns that extend along either side of the gut [[6]], perhaps suggesting a more stereotyped pattern of ENS development than seen in mice. The migratory behavior and hierarchical relationships of cells within the well-structured ENCC columns of zebrafish and their comparison to cells within pLLP and the mouse gut will certainly be the subject of future studies.

## Do ENCCs ultimately determine the organization of neural networks in the gut?

As ENCCs migrate and colonize the gut, a subset of them start to differentiate initially into neurons and later on into glial cells. In the mouse, enteric neurogenesis continues for several weeks after birth, highlighting the ability of newly born enteric neurons to be incorporated into fully functional neural circuits. The neural networks of the mammalian gut are extremely complex and the rules that govern their assembly and spatial organization are far from clear. One approach to clarifying the cellular and molecular mechanisms that regulate the formation of functional neural circuits in the ENS is to study the organization of nascent neurite modules in the embryonic gut. This approach has met some success recently when Sasselli *et al*. [[7]] demonstrated that core components of the planar cell polarity (PCP) pathway are necessary for aligning neurites along the rostrocaudal axis of the gut. Mutations in this pathway resulted in a considerable degree of randomization of neurite orientation, which was associated with characteristic changes in the peristaltic activity of the intestine. Interestingly, despite the altered organization of neurites in PCP mutants, no obvious deficit in the colonization of the gut by NC cells was observed, arguing that migration of ENCCs is independent of neurite orientation. Consistent with this view, Young and colleagues argue that neurites in the gut follow a migratory path laid down by advancing ENCCs. In support of this view, placement of a group of ENCCs at the caudal end of an aneural gut segment from *Ret* mutant embryos resulted in rostral migration of ENCCs and the longitudinal arrangement of neurites that extended in the same direction. Intriguingly, this direction was maintained even after orally directed neurites met an advancing population of ENCCs that was placed at the opposite end. But if neurites simply follow the direction of ENCC migration, why do orally directed axons that encounter caudally migrating ENCCs continue to advance rostrally rather than randomizing their trajectory? Perhaps enteric neurites imbued with a migratory direction at an early critical stage of outgrowth by cognate ENCCs are unable to reroute upon encountering a caudally advancing group of NC cells. If this is indeed the case, it would be interesting to examine the cellular and molecular mechanisms that underpin the apparently irreversible commitment of axons of enteric neurons along the rostrocaudal and circumferential axes of the mammalian gut.

## What controls polarity in the developing ENS?

One of the most interesting aspects of the Young *et al*. paper is the suggestion that all the information required to drive key events in gut colonization by NCs and neural circuit development, such as the effective rostrocaudal advance of the migratory front and the organization and direction of neurite outgrowth, is intrinsic to ENCCs and largely independent of the polarity of gut mesenchyme. Does this indicate that the spatial distribution of neurotrophic factors produced by the gut wall have no effect on the migratory behavior of ENCCs and the growth cones of newly born enteric neurons? What about signaling molecules such as glial cell line-derived neurotrophic factor (GDNF), which is expressed by mesenchymal cells and, in addition to being critical for the survival and proliferation of ENS progenitors, functions as a guidance molecule for ENCCs *in vitro* [[8],[9]]? Or members of the Wnt family, many of which are expressed in the embryonic gut and are critical in organizing neural networks in other parts of the nervous system? We suggest that there is no contradiction between the self-organizing ability of ENCCs and the proposed roles of these or other gut mesenchyme-derived factors. ENCCs and neurites could themselves contribute to the generation of local gradients of widely expressed mesenchymal factors (such as GDNF) that in turn direct their migration. The self-determined directional migration of cells through the local redistribution of extracellular cues has been demonstrated experimentally in pLLP [[10]]. In this case, gradients of the widely distributed chemokine Cxcl12a are generated across its length via polarized internalization of Cxcl12a by the receptor Cxcr7 at the trailing end and activation of Cxcr4 in the leading edge to propel the primordium. It would be of great interest to explore the potential role of the GDNF receptors Gfra1 and Ret in the generation of local GDNF gradients and the self-directed formation of a uniform ENS plexus.

## Can gastrointestinal pathologies be traced to misbehaving ENCCs?

Shedding light on the cellular and molecular mechanisms that control the colonization of the intestine by neural crest cells is of obvious medical importance. A number of conditions are thought to result from deficits in the migration and ultimately the colonization of the gut by neural crest cells and the normal development of the ENS. Foremost among them is Hirschsprung’s disease, a congenital neurodevelopmental deficit of the ENS, which occurs in 1:4,500 live births and is characterized by absence of enteric ganglia from the distal colon. In addition, a number of ill-defined conditions, termed collectively as functional gastrointestinal disorders, are thought to originate from subtle changes in the connectivity of enteric neurons. Therefore, understanding the mechanisms that regulate the migration of neural crest cells and how they may influence the connectivity of enteric neurons is critical for deciphering the pathogenesis of a broad range of congenital and acquired deficits of enteric neural activity and digestive function.
